# Identification and Characterization of Circular RNAs in Association With the Deposition of Intramuscular Fat in Aohan Fine-Wool Sheep

**DOI:** 10.3389/fgene.2021.759747

**Published:** 2021-12-06

**Authors:** Le Zhao, Lisheng Zhou, Xiaojing Hao, Lei Wang, Fuhui Han, Lirong Liu, Xinming Duan, Feng Guo, Jianning He, Nan Liu

**Affiliations:** ^1^ College of Animal Science and Technology, Qingdao Agricultural University, Qingdao, China; ^2^ Qingdao Animal Husbandry and Veterinary Research Institute, Qingdao, China; ^3^ China Animal Health and Epidemiology Center, Qingdao, China; ^4^ Nongfayuan Zhejiang Agricultural Development Co. Ltd., Huzhou, China; ^5^ Tongliao Animal Agriculture Development Service Center, Tongliao, China

**Keywords:** aohan fine-wool sheep, intramuscular fat, regulatory mechanism, circular RNA, interaction analysis

## Abstract

Aohan fine-wool sheep (AFWS) is a high-quality fine-wool sheep breed that supplies wool and meat. Research is needed on the molecular mechanism behind intramuscular fat (IMF) deposition that greatly improves mutton quality. The widely expressed non-coding RNA is physiologically used in roles such as competitive endogenous RNA (ceRNA) that includes circular RNAs (circRNAs). Although circRNAs were studied in many fields, little research was devoted to IMF in sheep. We used the longissimus dorsi muscle of 2 and 12-month-old AWFS as research material to identify circRNAs related to IMF deposition in these sheep by RNA-seq screening for differentially expressed circRNAs in the two age groups. A total of 11,565 candidate circRNAs were identified, of which the 104 differentially expressed circRNAs in the two age groups were analyzed. Enrichment analysis was performed using Gene Ontology and the Kyoto Encyclopedia of Genes and Genomes. The enriched pathways included lipid transport (GO:0006869), negative regulation of canonical Wnt signaling pathway (GO:0090090), fat digestion and absorption (ko04975), and sphingolipid metabolism (ko00600). The differentially expressed circRNAs included ciRNA455, circRNA9086, circRNA7445, circRNA4557, and others. The source genes involved in these pathways might regulate IMF deposition. We used the TargetScan and miRanda software for interaction analysis, and a network diagram of circRNA-miRNA interactions was created. CircRNA455-miR-127, circRNA455-miR-29a, circRNA455-miR-103, circRNA4557-mir149-5p, and circRNA2440-mir-23a might be involved in the IMF deposition process. The targeting relationship of circRNA4557-miR-149-5p was verified by a dual-luciferase reporter assay. The RT-qPCR results of seven randomly selected circRNAs were consistent with the sequencing results. This study provides additional information on circRNA regulation of IMF deposition in AFWS and is a useful resource for future research on this sheep breed.

## Introduction

In recent years, with the improvement in the standard of living, the demand for meat products has increased. The IMF content is a factor determining meat quality. The quality of mutton sheep is affected by genetics and the environment (Warner et al., 2010; Gkarane et al., 2019), with the genetic part determining the selection progress between generations. Aohan fine-wool sheep (AFWS) is a sheep breed independently bred in China. The AFWS significantly contributes to mutton production in China, especially in Inner Mongolia (Liu et al., 2001; Liu et al., 2013). The research on its genes is particularly important, as it could help improve its meat quality. Therefore, only by clarifying the factors that affect meat quality and gene regulation mechanisms can we make effective choices. Mutton’s quality is particularly affected by the IMF deposition (Scollan et al., 2017). The associated reaction mechanism is an intricate biological process, jointly regulated by epigenetic modifications and related gene expression (Fu et al., 2018; Mortimer et al., 2018). Therefore, IMF gene regulation research is of great significance. Current research shows that a variety of molecules are involved in IMF regulation. For example, a functional regulatory sequence variant was identified in pig *MYH3*, providing new insights into the IMF regulation mechanism in pigs (Cho et al., 2019). *PPARG*, *LPL*, *FABP4*, *THRSP*, *RBP7*, *PLIN*, and *LDLR* are responsible for IMF deposition in chicken (Cui et al., 2018). Many studies have investigated the IMF regulatory pathways, such as the sphingolipid signaling pathway (KEGG ssc04071) (Ding et al., 2019). The peroxisome proliferator activated-receptors (PPAR) signaling pathway was reported to be directly involved in fat deposition in the pectoralis major of chicken (Liu et al., 2017). Glycerolipid metabolism and the fatty acid degradation pathway might contribute to the differences in IMF deposition (Liu et al., 2019). Many studies have recently shown that circRNAs play an important role in IMF. Li et al. found that circRNAs influenced IMF in Donkeys (Li et al., 2020). Studies have also found that circRNA inhibits the differentiation of preadipocytes (Shen et al., 2021). CircRNA has also been shown to adjust IMF content and improve meat quality (Wang et al., 2020).

CircRNAs are endogenous covalently closed circular non-coding RNAs expressed in eukaryotic cells. They are formed by back-splicing of mRNA precursors (pre-mRNA) (Memczak et al., 2013; Chen and Yang, 2015; Cooper et al., 2018). The first covalently closed circRNA was discovered in 1976 in plant viruses (Sanger et al., 1976). It is different from the standard linear RNA, as its 3′ and 5′ ends are joined to form a covalently closed loop (Kristensen et al., 2019), which makes them more stable (Qu et al., 2015) and resistant to digestion by RNase R (Jeck and Sharpless, 2014; Xiao and Wilusz., 2019). In the early days, circRNA was assumed to have no function. After in-depth research, it was found that circRNA could perform its biological functions in various ways (Li et al., 2018). CircRNA can bind to RNA binding protein to regulate gene expression and protein translation, act as a miRNA sponge, encode functional proteins, and participate in cell communication and signal transduction (Tu et al., 2018). circRNA can be classified into the following four types according to their sources: 1). All-exon circRNA (Jeck et al., 2013); 2). Exon-intron circular RNAs (EI circRNAs) that combine introns and exons (Liu et al., 2013); 3). Lasso-type, composed of circular intronic RNA (ciRNA) (Li et al., 2017); 4). circRNA produced by the circularization of viral genome RNA, transfer RNA (tRNA), ribosomal RNA (rRNA), small nuclear RNA (snRNA), and more (Zhang et al., 2013). This last type belongs to the intergenic circRNA. Different types of circRNA play different biological functions.

Recent studies reported that circRNA plays a significant role in human visceral fat cells (Sun et al., 2020). CircRNA has also been studied extensively in mice and was reported to be involved in IMF deposition (Long et al., 2018). Some researchers have identified differentially expressed genes (DEGs) and circRNAs in the adipose tissue of buffaloes aged six and 30 months and the circRNA-miRNA interactions that regulate IMF deposition in them (Huang et al., 2019). Several competitive endogenous RNAs (ceRNAs) and many circRNAs related to adipocyte differentiation were identified in yak (Zhang et al., 2020). Studying 0.5 and 2.5-year-old yaks, the researchers constructed a ceRNA network based on identified mRNAs and circRNAs potentially involved in IMF deposition, laying the foundation for subsequent research (Wang et al., 2020). Nevertheless, there are few studies on the involvement of circRNAs in IMF deposition in sheep.

In a preliminary study, we evaluated the IMF content of 2, 4, 6, and 12-month-old AFWS. The results showed that the IMF content in the 2 and 12-month-old sheep was significantly different (*p* < 0.01) (Han et al., 2021). Therefore, in this study, we tested circRNAs expression in IMF-related tissues of 2-month-old (Mth-2) and 12-month-old (Mth-12) AFWS to analyze the association between circRNA and IMF changes at different developmental stages. Our results indicate that circRNAs are important regulators of sheep IMF deposition.

## Materials and Methods

### Sample Collection and Preparation

This study was performed on healthy 2-month-old (*n* = 3) and 12-month-old (*n* = 3). AFWS rams raised in an AFWS station in the Inner Mongolia Autonomous Region according to the farm’s feeding and housing plan. Their maternal age and weight were similar, and they underwent estrus and artificial insemination at the same time. We anesthetized the rams by intravenous injection of sodium pentobarbital at a dose of 25 mg/kg following published protocols (Raj et al., 2004; Hawkins et al., 2016), and then placed them in a closed room that was filled with carbon dioxide at a rate of 20% per minute. When the gas concentration had reached 80%, the rams had died. Samples of the longissimus dorsi muscle were collected, placed in Eppendorf tubes, and immediately stored in liquid nitrogen. The frozen samples were transported to the laboratory for subsequent testing.

### RNA Extraction and Quality Assessment

TRIzol reagent (Invitrogen, Carlsbad, CA, United States) extracted total RNA from the six samples. We then used Agilent 2100 Bioanalyzer (Agilent, Santa Clara, CA, United States), NanoDrop ND-2000 (Thermo Fisher Scientific, Waltham, MA, United States), and 1% agarose gel electrophoresis to determine the RNA quality and quantity in the samples. The RNA integrity number (RIN) value, based on A260/A280 and 28S/18S ratios, had to meet the high-throughput sequencing requirements to be used for subsequent sequencing analysis. The required A260/A280 range was 1.8–2.0. If the 28S and 18S bands were clear and with no impurities, the RNA quality was acceptable. If the level of 28S was about twice that of 18S, the sample RNA conformed to the standard required for library construction. After an appraisal, all samples met the requirements of library construction.

### Library Construction and CircRNA Sequencing

After total RNA extraction, rRNA depletion was used to construct a chain-specific library. Sequencing of the qualified library was done by Illumina Hiseq 4000 (LC Sciences, Houston, TX, United States), with a double-end sequencing read length of 2 × 150 bp (PE150). First-strand complementary DNA (cDNA) was synthesized using random hexamer primers and M-MuLV reverse transcriptase (RNase H-). The Second-strand cDNA was then synthesized with dNTPs, DNA polymerase I, and RNase H. Subsequently, T4 and Klenow DNA polymerases were used to repair and modify the ends. The cDNA products were then purified using AMPure XP beads (Beckman Coulter, Brea, CA, United States). Finally, uracil DNA glycosylase (NEB, Ipswich, MA, United States) was used to degrade the U-containing chains to remove the second-strand cDNA. The purified first-strand cDNA was enriched by PCR to obtain a cDNA library.

### Statistical Analysis and Quality Control of the Sequencing Data

We filter out unqualified sequences, including reads with adapters, reads that contained >5% N (N stands for undetermined base information), and low-quality reads (a basic group with a quality value of Q < 10). For base accounts of over 20% of the entire read, we quantified the original sequencing, effective sequencing, Q20, Q30, and GC content and conducted a comprehensive evaluation.

### Sequencing Analysis of CircRNA

We chose *Ovis aries* Ensembl 96 as the reference genome to compare with RNA-Seq data. We use the cutadapt 1.9 software to remove the linker sequence from the original data. We then removed repeated and low-quality sequences to obtain the clean data. We used the FastQC v0.10.1 software to perform quality control analysis on the clean data and used HISAT2-2.0.4 to compare the obtained clean data to the genome. We used TopHat-Fusion to align unaligned sequences and used CIRCExplorer2 v2-2.2.6 and CIRI v2.0.2 to predict circRNA (Dong et al., 2019). Subsequently, the circRNA was quantified and normalized.

### CircRNA Differential Expression Analysis

The expression level of circRNA was normalized using spliced reads per billion mappings (SRPBM). We used three biological replicates for each age group. The screening conditions used when analyzing the identified circRNAs differential expression were |log2 Fold Change| ≥ 1 and *p* ≤ 0.05. Multiple screening conditions were comprehensively set during analysis to obtain the number of up- and downregulated circRNAs.

### GO and KEGG Enrichment Analyses

Gene Ontology (GO) (http://www.geneontology.org) has three major categories: molecular function, cell composition, and biological process (Ashburner et al., 2000). The Kyoto Encyclopedia of Genes and Genomes (KEGG) (http://www.genome.ad.jp/kegg/) can be used to query metabolic pathways (Xia et al., 2016), enzymes (or enzyme-coding genes) (Kanehisa, 2017), and metabolites (Navarro et al., 2019). The differentially expressed circRNAs were annotated using GO and the KEGG pathway analyses. GO function analysis was performed by the Blast2GO method (Conesa and Götz, 2008), and statistical enrichment of differential gene expression in the KEGG pathway analysis was detected by the KOBAS software (Götz et al., 2008).

### Interaction Analysis of CircRNA and MiRNA

Two software programs, TargetScan and miRanda, were used to predict the targeting relationship between circRNAs and miRNAs (Da Costa Martins and De Windt, 2012). TargetScan performs miRNA target prediction based on the seed region, while miRanda is based mainly on the free energy of the circRNA and miRNA. The lower the free energy, the stronger the binding capacity of the two. Therefore, we chose TargetScan and miRanda to predict the target relationship between the circRNAs and miRNAs.

### Dual-Luciferase Reporter Assay

The targeting relationship between circRNA4557 and miR-149-5p was verified by the dual-luciferase reporter assay. Wild type (WT) and mutant vector (MUT) of circRNA4557 were cloned. These constructs were co-transfected into 293T cells with miR-149-5p mimetic or negative control. A lysis mixture (20 µl) was added to the sample, followed by 100 µl of luciferase assay reagent II (LARⅡ). The renilla luciferase activity acted as an internal control, and 100 µl of Stop and Glo reagent (Promega) was added to determine the luciferase activity of the sea pansy (*Renilla reniformis*) reporter gene.

### Experimental Verification by RT-qPCR

We randomly selected seven circRNAs and verified their expression levels by RT-qPCR. We used Evo M-MLV RT Kit with gDNA Clean for qPCR II (AG, Changsha, Hunan, China) to convert the total RNA to cDNA. The RT-qPCR analysis was carried out in triplicate on Bio-Rad CFX96 (Bio-Rad, CA, United States). *GAPDH* was used as an internal reference. A reaction volume of 20 μl contained 10 μl SYBR Green Premix Pro Taq HS qPCR Kit (AG, Changsha, Hunan, China), 1.6 μl cDNA, 7.6 μl ddH_2_O, and 0.4 μl each of the forward and reverse primers. Primer sequences are listed in Supplementary Table S1. The RT-qPCR reaction conditions were set as follows: 95°C for 30 s; 40 cycles of 95°C for 5 s, 58°C for 30 s; 65°C for 30 s; the final stage was at 95°C for 5 min. The 2^−ΔΔCt^ method was used to analyze the relative expression levels of the selected circRNAs.

Total RNA and RNase R (Geneseed Biotech, Guangzhou, Guangdong, China) were mixed to determine the resistance of the selected seven circRNAs to RNase R digestion. The mix was incubated at 37°C for 15 min cDNA was then synthesized, and RT-qPCR assessed the expression level of the selected circRNAs.

### Statistical Analysis

Data on IMF content are expressed as means ± standard deviation. SPSS Statistics for Windows, Version 17.0 (SPSS Inc., Chicago, IL, United States) was used to analyze the experimental results using one-way analysis of variance. The RNA-seq results were analyzed by the SPSS Statistics for Windows, Version 17.0 and R software programs (*p* < 0.01 means extremely significant, *p* < 0.05 means significant).

## Results

### Determination of IMF Content at Mth-2 and Mth-12 Sheep

The research measured the IMF in the longissimus dorsi of Mth-2 and Mth-12 sheep. The IMF content in the Mth-12 sheep (11.2 ± 0.9%) was significantly higher than in Mth-2 sheep (2.2 ± 0.006%; *p <* 0.01).

### Sequencing and Localization of the CircRNA in Sheep Muscle

**FIGURE 1:**
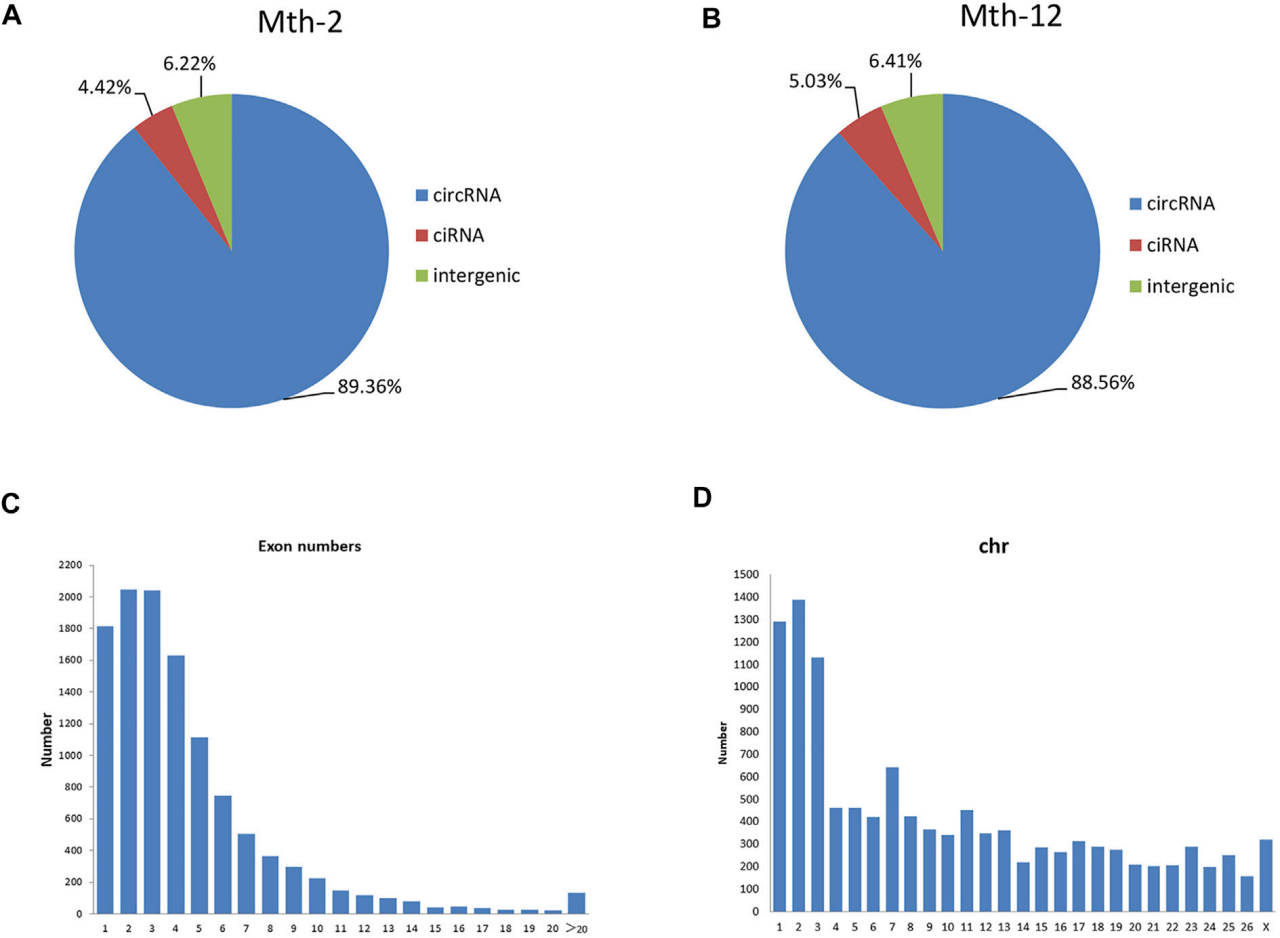
General characteristics of Aohan fine-wool sheep (AFWS) circular RNAs (circRNAs); **(A)** The types of circRNAs in 2-month-old (Mth-2) rams; **(B)** The types of circRNAs in 12-month-old (Mth-12) rams; **(C)** The number of exons; **(D)** Distribution of circRNAs on the chromosomes.

The preliminary filtered data are listed in Supplementary Table S2. The main circRNA types identified in this study were all-exon (Mth-2: 89.36%; Mth-12: 88.56%), lasso-type circRNAs (Mth-2: 4.42%; Mth-12: 5.03%), and the intergenic type (Mth-2: 6.22%; Mth-12: 6.41%; Figures 1A,B). CircRNAs usually consist of one to four exons (Figure 1C). It has been reported that the length of circRNAs with only one exon was longer than that of circRNA with multiple exons (Tan et al., 2018). The number of circRNAs detected varied between chromosomes, with most localized on chromosomes 1, 2, and 3 (Figure 1D).

### Expression Analysis of CircRNA in Muscle Tissue

**FIGURE 2:**
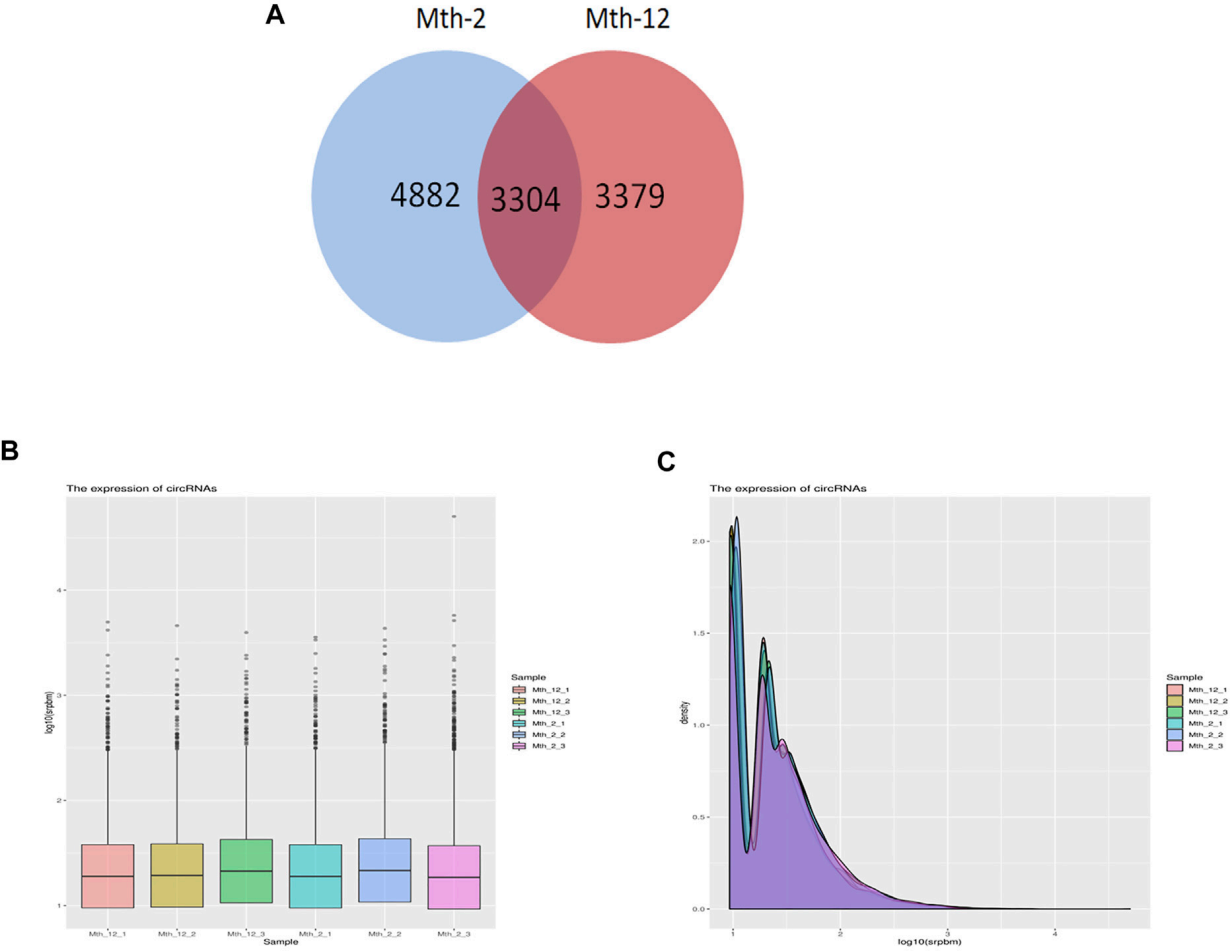
CircRNA expression. **(A)** Venn diagram, drawn using the number of circRNAs in the two age groups; **(B)** circRNA expression; **(C)** The peak density of circRNA expression.

A total of 11,565 candidate circRNAs were identified. Mth-2 samples expressed 8,186 circRNAs, and Mth-12 samples expressed 6,683. Of these, 3,304 circRNAs were expressed in both age groups (Figure 2A). The 30 circRNAs with the highest expression levels are listed in Table 1, and the types of circRNA are marked. circRNA2800, circRNA2441, circRNA328, and ciRNA67 had the highest expression level in two age groups. Based on all the above, we speculate that these four circRNAs regulate IMF deposition in AWFS. Only a few circRNAs belonged to the circular intronic RNA (ciRNA) and intergenic types. The circRNAs expression is shown in Figure 2B. It can be seen from Figure 2C that the peak gene density in association with circRNA expression was between 0.75 and 1.5.

**TABLE 1:** The top 30 expressed circRNAs in Mth-2 and Mth-12 rams.

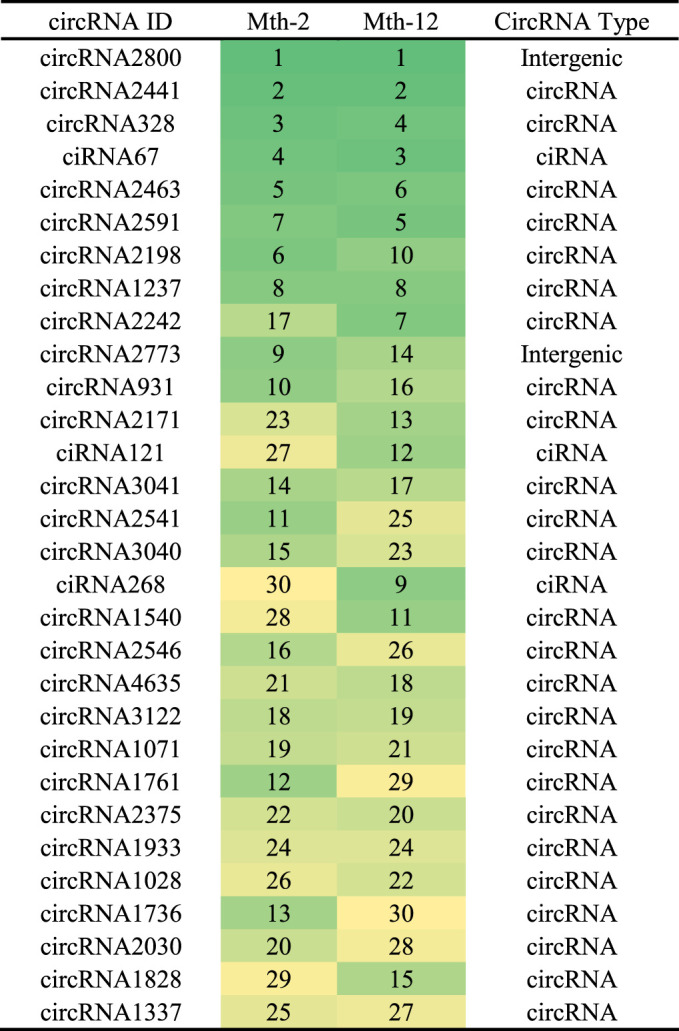

Green means higher expression and yellow means lower expression. The overrepresentation of green and yellow goes from high to low. The numbers from 1 to 30 in each column represent the descending order of circRNA, expression levels.

### Identification of Differentially Expressed CircRNAs

**FIGURE 3:**
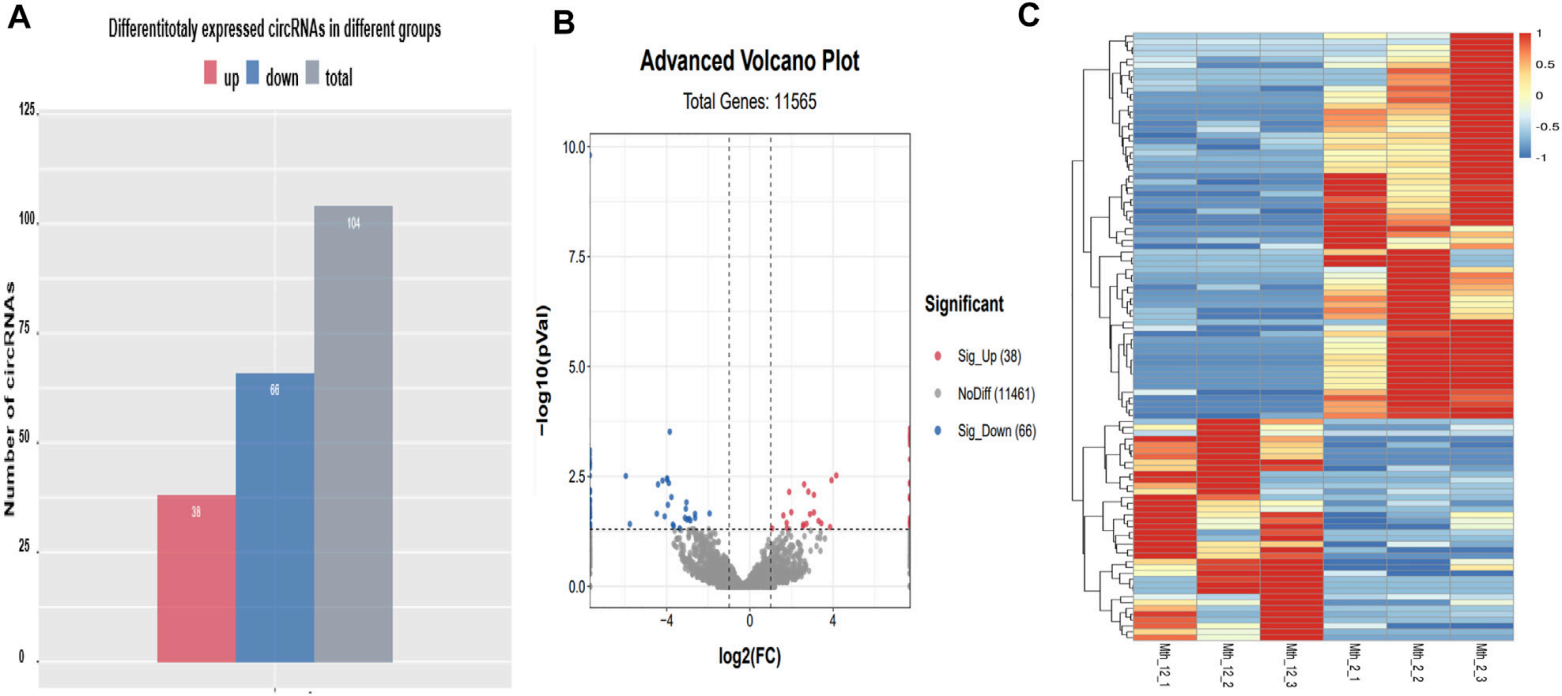
Differentially expressed circRNAs. **(A)** A histogram of differentially expressed circRNAs; **(B)** A scatter plot of differentially expressed circRNAs; **(C)** A cluster heat map of differentially expressed circRNAs. The abscissa is the sample, and the ordinate is the differentially expressed screened-out genes. Different colors indicate different gene expression levels. The color changes from blue to white to red, with red indicating highly expressed genes and blue indicating genes with low expression levels.

Among the 104 differentially expressed circRNAs detected (Figure 3A), 38 were upregulated, and 66 were downregulated. The overall distribution of the differentially expressed circRNAs is visually presented as a scatter plot in Figure 3B and as a cluster heat map in Figure 3C. The related data are listed in Supplementary Table S3. Among the differentially expressed circRNAs, Titin (*TTN*) is the source gene of circRNA3117, circRNA7648, circRNA4474, circRNA153, circRNA1817, and circRNA3096. The circRNA3096 was differentially expressed, but found in high levels in both age groups, suggesting it is active and participates in IMF growth and development during the entire period. We found that the source gene of circRNA2639 was *USP34*. *USP34* plays a critical role in the Wnt/β-catenin signaling pathway. We will study these circRNAs in the future as they might be related to IMF.

### Differentially Expressed CircRNA-Hosting Genes Based on GO Enrichment Analysis

**FIGURE 4:**
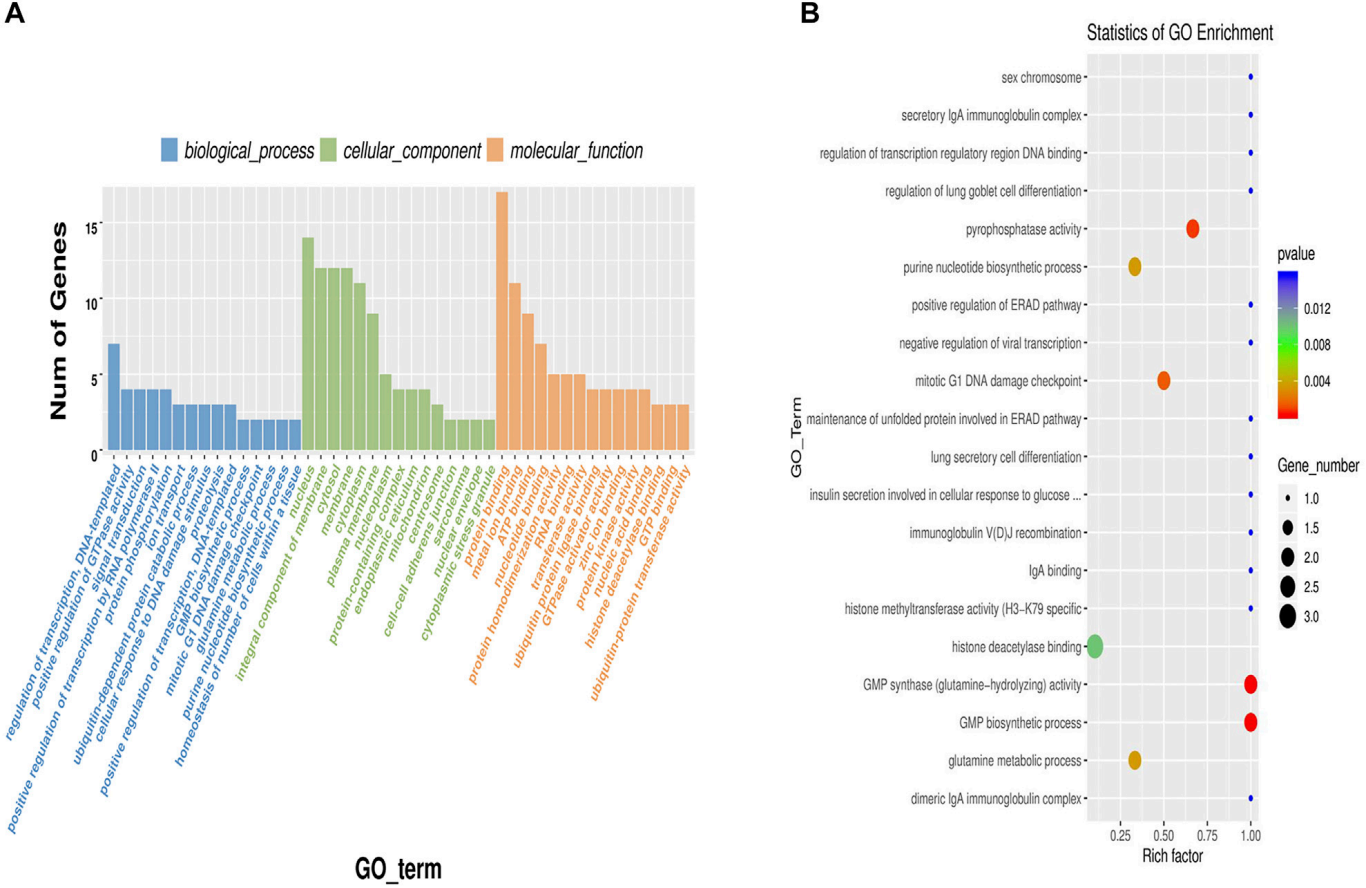
Gene Ontology (GO) enrichment analysis of differentially expressed circular RNA (circRNA)-hosting genes. **(A)** A histogram of differentially expressed circRNA-hosting genes enrichment analysis; **(B)** A scatter plot of differentially expressed circRNA-hosting genes enrichment analysis. The dot size represents the number of genes with a significant difference that matches the S gene number of a single GO term, and the color of the dot represents the *p*-value of the enrichment analysis (i.e., enrichment significance).

Gene Ontology (GO) enrichment analysis found 251 enriched groups in the biological process classification. The most significantly enriched group was regulation of transcription, DNA-templated (GO:0006355), followed by positive regulation of GTPase activity (GO: 0043547), signal transduction (GO:0007165), positive regulation of transcription by RNA polymerase II (GO:0045944), and protein phosphorylation (GO:0006468). Some GO terms related to fat metabolism were enriched, including lipid transport (GO:0006869), negative regulation of canonical Wnt signaling pathway (GO:0090090), and lipid metabolic process (GO:0006629). Some circRNAs and their source genes were significantly enriched. CircRNA2666 and its source genes were significantly enriched in GO:0006869. CircRNA6495 and its source genes were significantly enriched in GO:0090090. And circRNA455 and its source genes were significantly enriched in GO:0006629. CircRNA2666, circRNA6495, and circRNA455 are important candidate genes. We sorted the classifications in descending order according to the number of differentially expressed genes annotated by each GO term (S gene number) and selected the top 15 in all three classifications to draw a histogram (Figure 4A). We also took the 20 significantly enriched GO terms (*p*-value) to draw a scatter plot (Figure 4B).

### Differentially Expressed CircRNA-Hosting Genes Based on the KEGG Enrichment Analysis

**FIGURE 5:**
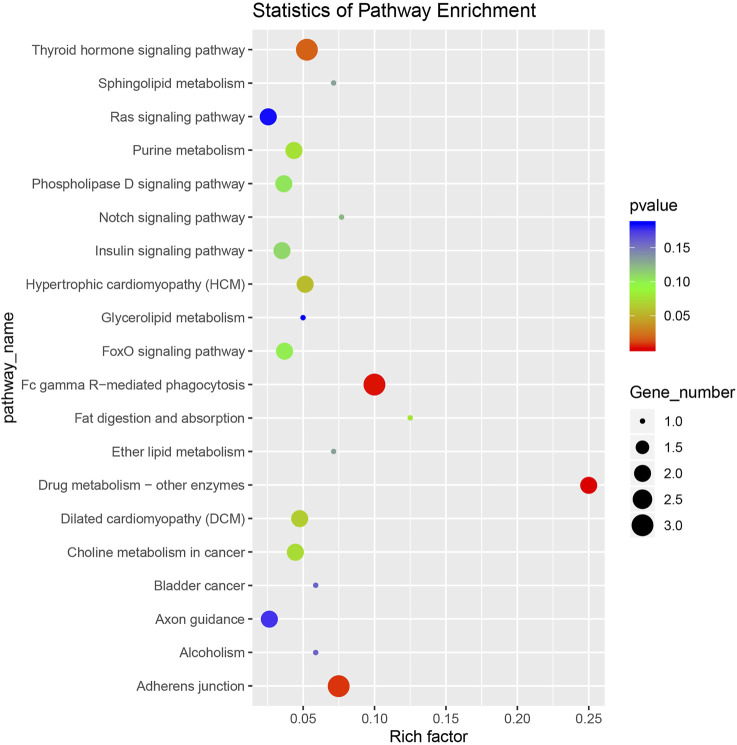
A scatter plot of the Kyoto Encyclopedia of Genes and Genomes (KEGG) enrichment analysis of differentially expressed circRNA-hosting genes.

Ninety-five pathways were significantly enriched in the KEGG enrichment analysis. We took the top 20 significantly enriched KEGG terms (*p*-value) and drew a scatter plot (Figure 5). The most abundant gene enrichment pathways were Fc gamma R-mediated phagocytosis (ko04666), adherens junction (ko04520), and thyroid hormone signaling pathway (ko04919).

Based on the KEGG enrichment analysis, we identified pathways with more enriched genes that might be related to IMF deposition. These included fat digestion and absorption (ko04975), Notch signaling pathway (ko04330), sphingolipid metabolism (ko00600), ether lipid metabolism (ko00565), glycerolipid metabolism (ko00561), glycerophospholipid metabolism (ko00564), glutamatergic synapse (ko04724), MAPK signaling pathway (ko04010), and sphingolipid signaling pathway (ko04071). We assembled 12 signaling pathways and list their enriched source genes and corresponding circRNAs in Supplementary Table S4. CiRNA455 and its source gene were significantly enriched in ko04666, ko04975, ko00600, ko00565, ko00561, and ko00564. Therefore, we speculate that ciRNA455 regulates the deposition of IMF. CircRNA7445 and its source gene *KAT2B* were significantly enriched in two signaling pathways, ko04919 and ko04330. And *KAT2B* was reported to affect carcass fat deposition in beef cattle (Moisá et al., 2013). These circRNAs could be considered important candidate circRNAs, affecting IMF deposition in sheep during the later growth stage.

### Construction of CircRNA-miRNA Interaction Network

**FIGURE 6:**
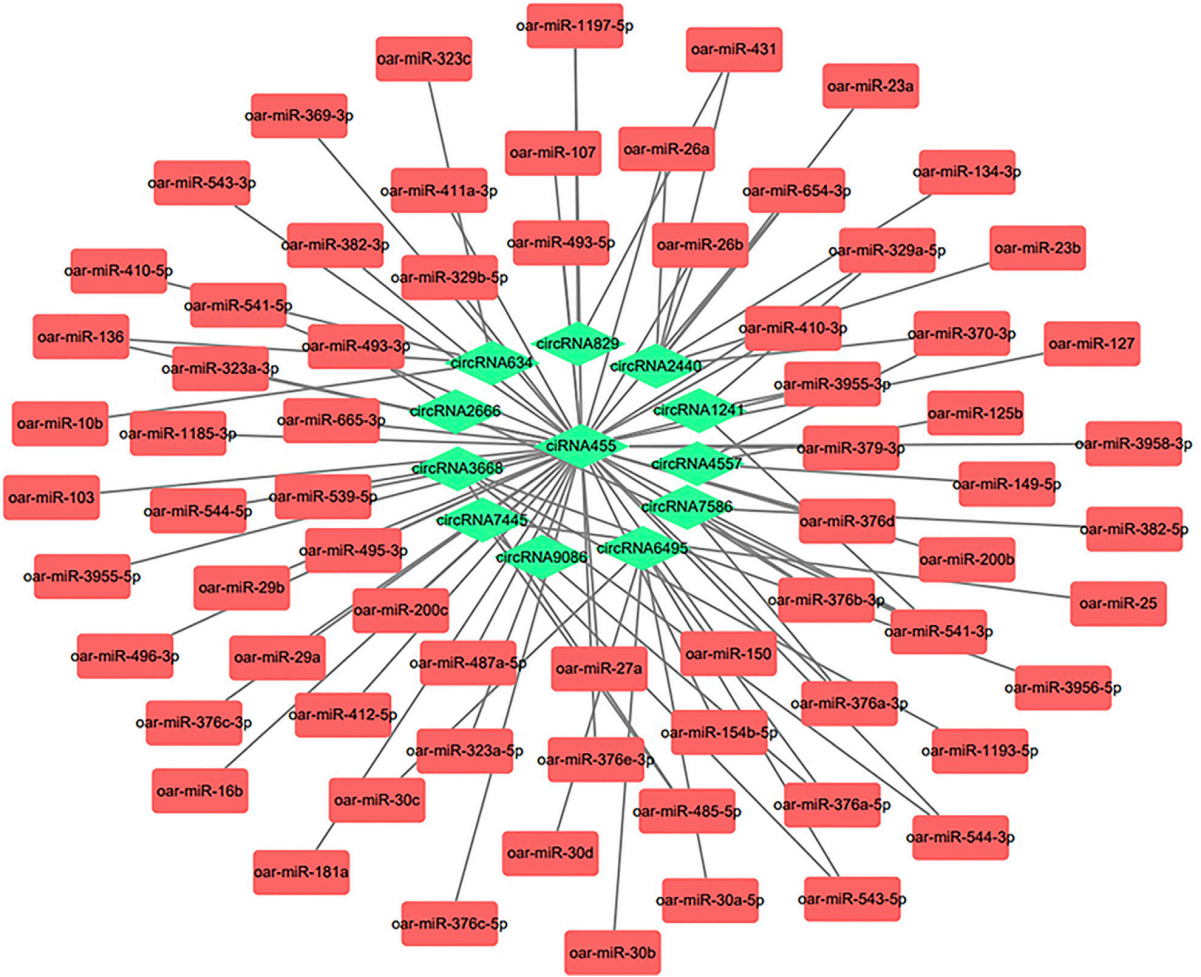
Interaction analysis network diagram of circular RNAs (circRNAs) and micro RNAs (miRNAs). The circRNA-miRNA network diagram was constructed based on 12 circRNAs and 70 miRNAs.

CircRNAs could act as a miRNA sponge, thus inhibiting the miRNAs from binding to their target genes (Wilusz and Sharp, 2013). We selected ten candidate circRNAs that might be related to IMF deposition based on the differential expression, GO, and KEGG analysis results (circRNA2666, circRNA6495, ciRNA455, circRNA9086, circRNA2440, circRNA3668, circRNA829, circRNA634, circRNA7445, circRNA7586, circRNA1241, and circRNA4557). The miRanda software predicted 70 target miRNAs for the 12 candidate circRNAs. We also used the Cytoscape software to build a relationship network between them (Figure 6 and Supplementary Table S5). We identified 48 interactions, including circRNA455-miR-127, circRNA455-miR-29a, and circRNA455-miR-103. Interestingly, a circRNA could target multiple miRNAs. According to the prediction, ciRNA455 could target many miRNAs. A target relationship was found for circRNA4557-mir149-5p and circRNA2440-mir-23a. CiRNA455, circRNA4557, and circRNA2440 were significantly enriched in fat-related GO and KEGG pathways. We suspect that these circRNAs are related to IMF deposition. We will study these five circRNAs-miRNAs interactions in the future.

### Verification of Target Relationship for CircRNA4557-miR-149-5p

**FIGURE 7:**
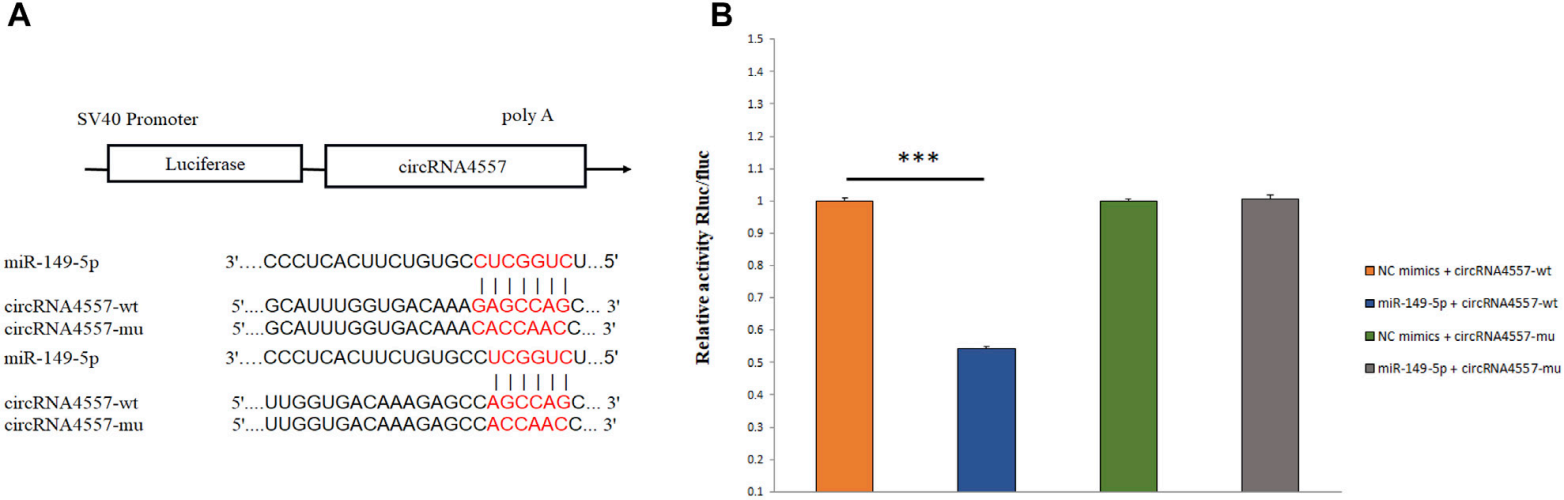
Detection of targeting relationship by dual-luciferase reporter assay. **(A)** A schematic diagram of miR-149 binding to circRNA4557 target site; **(B)** Detection of the interaction between miR-149-5p and circRNA4557 by dual-luciferase reporter assay.

We randomly selected circRNA4557-miR-149-5p to verify the targeting relationship using dual-luciferase reporter assay in the circRNA-miRNA regulatory network. We predicted the targeted binding site between circRNA4557 and miR-149-5p, and the two have the potential to bind (Figure 7A). And verified by Dual-luciferase reporter assay. Dual-luciferase reporter assay showed that miR-149-5p significantly reduced the luciferase activity by binding to the target site of circRNA4557 (*p* < 0.01; Figure 7B). The results indicated that circRNA4557 had a target relationship with miR-149-5p. It was speculated that circRNA4557-miR-149-5p played a role in IMF deposition in sheep.

### Verification of CircRNA Expression Level

**FIGURE 8:**
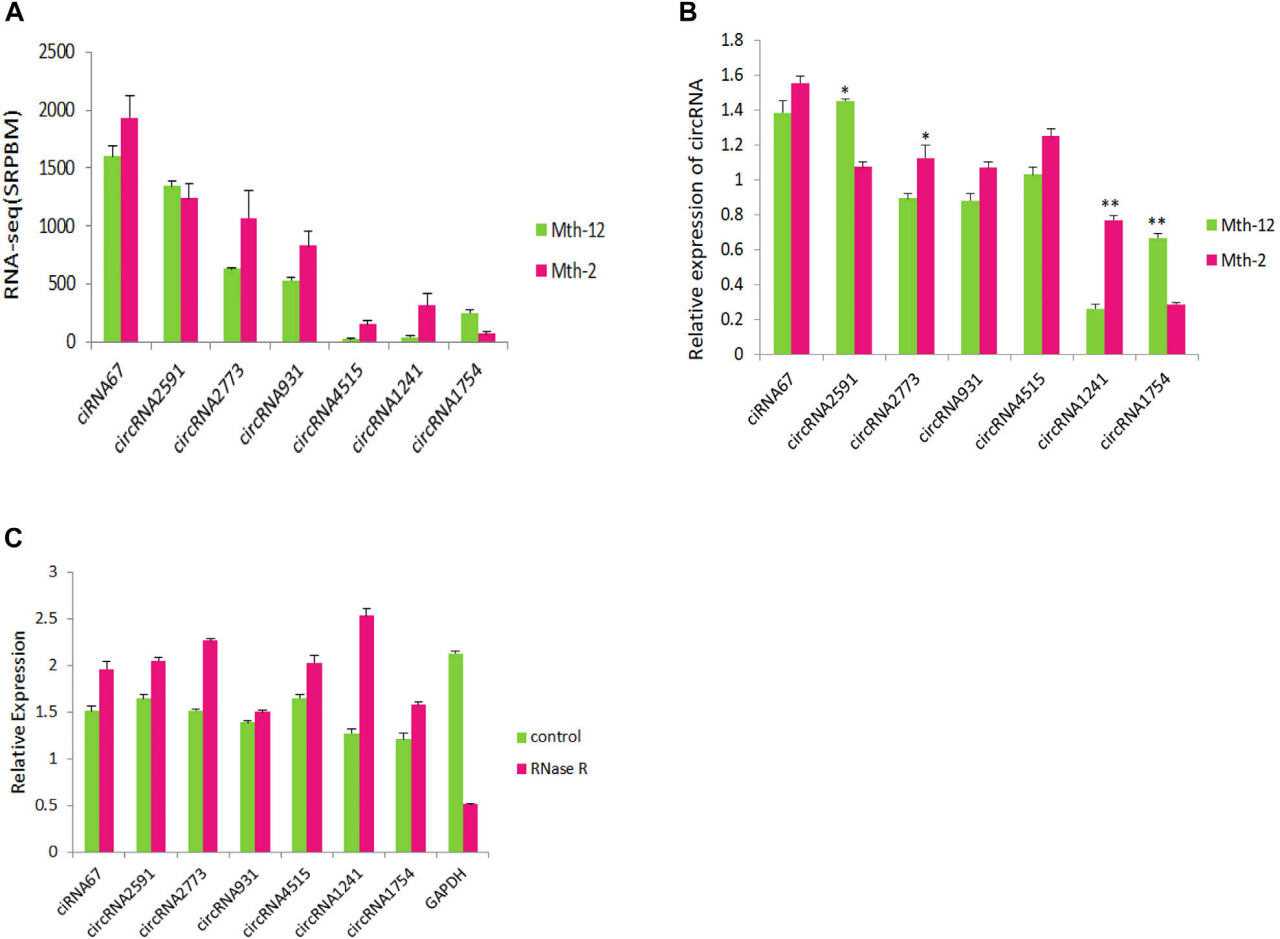
Verification of circular RNAs (circRNAs) and resistance to RNase R. **(A)** Sequencing results of seven circRNAs; **(B)** Reverse transcriptase quantitative real-time PCR (RT-qPCR) verification results of seven circRNAs; **(C)** The expression levels of circRNAs, and *GAPDH* after RNase R treatment.

We randomly selected seven circRNAs and detected their expression levels by reverse transcription quantitative real-time PCR (RT-qPCR; Figure 8A). The results were consistent with the trends observed in the RNA-Seq data (Figure 8B). This suggested that the RNA-Seq analysis was reliable. As shown in Figure 8, the selected circRNAs resisted RNase R digestion, while linear RNA (*GAPDH*) in the samples did not. RT-qPCR performed after RNase R digestion showed that the relative expression level of the seven circRNAs did not decrease significantly. Instead, in most, it has actually increased. The results showed that circRNA could resist digestion by RNase R, while linear RNA could not.

## Discussion

Sheep meat quality and its IMF content have a great influence on its taste (Lambe et al., 2017). Therefore, it is necessary to study the molecular mechanism behind IMF deposition. For the first time, the expression of circRNAs in sheep IMF in two age groups was studied. We detected circRNAs that might have a regulatory relationship with IMF deposition, and constructed a regulatory network based on this information.

The analysis relied on the 104 differentially-expressed circRNAs detected in our study. The differential expression of the circRNA7819 and circRNA2959 genes was highly significant. Based on all the above, we speculate that these circRNAs play a role in the IMF regulatory mechanism in the two studied age groups. Among the differentially expressed circRNAs, circRNA4557 and the source gene *MSRB3* contributed to fat deposition in cattle (Wu et al., 2019). The source of circRNA3117, circRNA7648, circRNA4474, circRNA153, circRNA1817, and circRNA3096 was the Titin (*TTN*) gene. Researchers found that *TTN* could affect fat deposition in beef cattle muscle (Sasaki et al., 2006; Lyubetsky et al., 2017), possibly by exerting its effect on adipocyte-lineage cells or the milieux surrounding them. The source gene of circRNA2639 identified in this study is *USP34*, which plays a critical role in the Wnt/β-catenin signaling pathway (Oh et al., 2017; Lui et al., 2011). We analyzed the interaction between circRNA, miRNA, and mRNA in IMF regulation. The source gene of circRNA7051, *SMC6*, was associated with fat metabolism in humans Ca (Camargo et al., 2013). The source gene of circRNA829 is Sorbin and SH3 domain containing 1 (*SORBS1*). Studies have reported that *SORBS1* is related to fat deposition in muscles (Sasaki et al., 2006). Therefore, we speculate that the DEGs of circRNA4557, circRNA3117, circRNA7648, circRNA4474, circRNA153, circRNA1817, circRNA3096, circRNA7051, circRNA829, and circRNA2639 might play a role in the regulatory mechanism of IMF.

We conducted GO enrichment analyses on the differentially expressed circRNA-hosting genes. Previous studies have shown that many pathways are related to the deposition of fat. In our research, circRNA6496 and its source gene were enriched in negative regulation of canonical Wnt signaling pathway (GO:0090090). The Wnt signaling pathway was related to bovine longissimus dorsi muscle IMF content (Jeong et al., 2013) and was shown to be a negative regulator of adipogenesis (Luo et al., 2015). According to relevant reports, lipid transport (GO:0006869), lipid metabolic process (GO:0006629) were important GO pathway related to fat deposition (Oliveira et al., 2018). CircRNA2666 and its source genes were enriched in GO:0006869, and circRNA455 and its source gene were enriched in GO: 0006629. The other circRNAs and their source genes were also significantly enriched in GO terms related to IMF characteristics. Based on all the above, the report speculate that circRNA6496, circRNA 2666, and circRNA455 are related to the IMF regulation mechanism.

The KEGG is a set of databases and related software used to extract genomic information (Kanehisa, 2002). It integrates information about molecules, genes, proteins, and biochemical compounds, and related reactions (Kanehisa et al., 2004). We analyzed the differentially expressed source genes of these circRNAs based on KEGG functions. The enriched pathways related to fat deposition were digestion and absorption (ko04975), sphingolipid metabolism (ko00600), ether lipid metabolism (ko00565), glycerolipid metabolism (ko00561), glycerophospholipid metabolism (ko00564), and other KEGG terms that might be related to fat metabolism. CiRNA455 was enriched in all these KEGG pathways. CircRNA7445 and its source gene K (lysine) acetyltransferase 2B (*KAT2B*) were significantly enriched in two signaling pathways (ko04919 and ko04330). The source gene of circRNA2997 is spermatogenesis-associated protein-7 (*SPATA7*). These source genes have been reported to be related to fat deposition (Sato et al., 2006; Moisá et al., 2013). Based on all the above, we speculate that ciRNA455 and circRNA7445 might be related to IMF deposition.

CircRNA was shown to act as a sponge for miRNA (Panda, 2018). In our research, many circRNAs could bind to miRNAs, and the two worked together to play a role in the IMF regulation mechanism in AWFS. Some circRNAs have multiple binding sites for miRNAs. According to the KEGG and GO enrichment analysis and the results of the differentially expressed genes, we selected 12 important circRNAs that might be related to IMF deposition and predicted their target miRNAs. These results provided a theoretical basis for studying the regulatory relationship between circRNA and sheep IMF deposition. In our study, ciRNA455 could target 48 miRNAs, including miR-29a and miR-127. CiRNA455 and its target gene were significantly enriched in the lipid metaphysical process (Go:0006629), phosphatidate phosphatase (Go:0008195), and other pathways related to fat deposition. It was found that miR-29a negatively regulated the differentiation of porcine adipocytes (Wu et al., 2021), and miR-127 was identified as an inhibitor of porcine adipogenesis (Gao et al., 2019). Based on all the above, we speculate that ciRNA455-miR-29a and ciRNA455-miR-127 impact IMF deposition by forming a targeting relationship. CircRNA4557 could form a targeting relationship with miR-149-5p. It has been reported that miR-149-5p could inhibit the proliferation and differentiation of bovine adipocytes (Khan et al., 2020). In the future, circRNA4557-miR-149-5p will be an important candidate pathway to study in AWFS.

CircRNA9086, ciRNA455, circRNA2440, circRNA3668, circRNA829, circRNA634, circRNA7445, circRNA7586, circRNA1241, circRNA6978, and circRNA4557 are candidate circRNAs that will be used in our future research on the molecular regulation of IMF deposition. CircRNA455-miR-127, circRNA455-miR-29a, circRNA455-miR-103, circRNA4557-mir149-5p, and circRNA2440-mir-23a might be involved in the deposition process of IMF. Based on our results, further work is needed to reveal the molecular mechanism behind circRNA regulation of IMF deposition in AFWS.

## Conclusion

Intramuscular fat has an important influence on meat quality. Our study identified 104 differentially expressed circRNAs during the growth and development of intramuscular fat in Aohan fine-wool sheep. The KEGG enrichment analysis of differentially expressed genes identified 12 pathways that might be related to IMF deposition. Ten circRNAs and their source genes were enriched in these 12 pathways. By predicting the relationship between circRNA and miRNA, we constructed a circRNA-miRNA network. According to reports, many miRNAs, including miR-29a, miR-127, and miR-149-5p, are related to the regulation of fat. CiRNA455-miR-29a, ciRNA455-miR-127, and circRNA4557-miR-149-5p might be involved in the IMF deposition process. According to the dual-luciferase reporter assay, the circRNA4557-miR-149-5p pair has a targeting relationship. These results could help to further study the molecular mechanism of sheep IMF deposition.

## Data Availability

The RNA-Seq data were submitted to the SRA database under accession number SRR12247890. Additional data can be found in the Supplementary Material.

## Glossary

AFWS: Aohan fine-wool sheep
IMF: intramuscular fat
ceRNAs: competitive endogenous RNAs
pre-mRNA: mRNA precursors
tRNA: transfer RNA
rRNA: ribosomal RNA
snRNA: small nuclear RNA
EI circRNAs: exon-intron circular RNAs
ciRNAs: circular intronic RNAs
RT-qPCR: reverse transcriptase quantitative real-time PCR
Mth-2: 2-month-old
Mth-12: 12-month-old
miRNA: micro RNA
lncRNA: long non-coding RNA
circRNA: circular RNA
GO: Gene Ontology
The KEGG: The Kyoto Encyclopedia of Genes and Genomes
DEG: differentially expressed gene
PPARG: peroxisome proliferator-activated receptor gamma
LPL: lipoprotein lipase gene
FABP4: fatty acid binding protein 4
THRSP: thyroid hormone responsive
RBP7: retinol binding protein 7
CDH5: cadherin 5
NPR2: natriuretic peptide receptor 2
SOCS3: suppressor of cytokine signaling 3
H-FABP: heart fatty acid-binding protein
A-FABP: adipocyte fatty acid-binding protein
ACAA2: acetyl-COA acyltransferase 2
CLCN3: chloride channel, voltage-sensitive 3
USP34: ubiquitin-specific protease 34
SORBS1: sorbin and SH3 domain containing 1
KAT2B: K (lysine) acetyltransferase 2B
SPATA7: spermatogenesis-associated protein-7
MMP9: matrix metallopeptidase 9
RIN: RNA integrity number
SRPBM: spliced reads per billion mapping.
